# Editorial: Optic pathway glioma: A multidisciplinary entity, posing dilemmas in diagnosis and management

**DOI:** 10.3389/fsurg.2022.995404

**Published:** 2022-08-03

**Authors:** Zohreh Habibi, Ali Tayebi Meybodi, William B. Lo, Nelci Zanon

**Affiliations:** ^1^ Children’s Medical Center Hospital, Tehran University of Medical Sciences, Tehran, Iran; ^2^ New Jersey Medical School, Rutgers, The State University of New Jersey Newark, Newark, NJ, United States; ^3^ Department of Neurosurgery, Birmingham Children’s Hospital, Birmingham, United Kingdom; ^4^ Department of Neurosurgery, Federal University of São Paulo, São Paulo, Brazil

**Keywords:** optic pathway gliomas, multidisciplinary, low grade glioma, tissue sampling, targeted therapies

Editorial on the Research Topic

Optic pathway glioma: A multidisciplinary entity, posing dilemmas in diagnosis and management

by Habibi Z, Meybodi AT, Lo WB and Zanon N. (2022), Front. Surg. 9:995404. doi: 10.3389/fsurg.2022.995404

The optimum management of optic pathway gliomas (OPGs) has remained controversial for many years and over several generations of neurosurgeons. The scientific societies of various disciplines, including neurosurgery, neuro-ophthalmology, neuroradiology, neuro-oncology, and neuropsychology, have conducted sophisticated studies and taken serious efforts to shed light on this gray zone of pediatric neuro-oncology. Despite these efforts, no consensus is available for diagnostic approaches, follow-up algorithms, outcome predictors, and treatment modalities for OPGs (1). Besides, there is no convincing evidence for the association of clinical or radiological features with tumor progression. Even so, intracranial hypertension and lower age at diagnosis have been proposed as the risk factors of worse prognosis (2). The association with Neurofibromatosis Type I (NF1) and the management of NF1-related OPG is another controversial issue (3). The literature suggestions for the treatment of OPG range from a close observation of patients who are minimally symptomatic or non-symptomatic to chemotherapy as the accepted first-line of treatment in symptomatic patients (4). Although surgical interventions are suggested to be reserved for conditions such as hydrocephalus, tumor progression despite chemotherapy, and complete visual loss accompanied by proptosis, the role of tissue sampling continues to be debated (5, 6).

In the current research topic, a number of papers have been published by specialists from different disciplines, aiming to focus on different aspects of the multidisciplinary management of OPG. The intent was to provide a contemporary update on all issues, including the diagnostic methods, genetic investigations, evolution of care standards, algorithms to guide therapeutic management, and proposed surveillance timetable. Moreover, current evidence regarding the outcome of treatments and recent recommendations was also reviewed to raise awareness about the role of surgery in the management of OPG.

A brief overview of the papers in this issue signified that the optimal management of pediatric OPG is still challenging. Nonetheless, the very concept of management, especially treatment paradigm, is evolving unceasingly. At present, most centers provide individualized algorithms to achieve the best outcomes and minimize long-term disability. As the first line of treatment, chemotherapy has been shown to provide better survival benefits when applied as a combined treatment rather than mono-modality treatment (J.X. Lim, et al.). The role of surgical intervention, specifically for diagnostic purposes, is being intensely debated. Less than a decade ago, it was argued that being deep in the white matter tract reduces the possibility of performing a safe tissue sampling unless there is a large extrinsic component (7). Nowadays, the landscape of opportunities for potential benefits from targeted therapies has changed this attitude (O. Barinfeld, et al.) A better understanding of the biology of pediatric OPGs and its implications for the development of biological treatment has made operative tissue sampling a critical component of therapeutic planning, particularly in non-NF1 patients (D.C. Samples, et al.). Moreover, it is suggested that further studies on genetic and molecular panels may provide information for predicting dismal events such as apoplexy in high-risk patients (S. Baeesa, et al.). Indeed, to accomplish a strong multidisciplinary approach, new data advocate the possibility of tissue sampling in almost all cases, with acceptable morbidity (G. Del Baldo, et al.).

The last issue is the optimized long-term care of children suffering from OPG in association with NF1. Since NF1-related OPGs have more indolent courses and better prognosis, visual outcomes are a significant management challenge in these cases, particularly in patients of younger age. Children with retrochiasmatic tumors and those who required chemotherapy were shown to have worse visual outcomes, although most NF1-related patients showed good overall recovery. Considering the more favorable course, recent evidence supports minimizing radiologic screening of asymptomatic NF1 patients (J.L. Zeid). The experience from a referral center showed that a multidisciplinary team approach, coordinated by a central specialty clinic, should become a routine component in the multidisciplinary management of these patients (L.-N. Lohkamp, et al.).
